# Sea Urchin Pigments: Echinochrome A and Its Potential Implication in the Cytokine Storm Syndrome

**DOI:** 10.3390/md19050267

**Published:** 2021-05-11

**Authors:** Tamara Rubilar, Elena S. Barbieri, Ayelén Gazquez, Marisa Avaro

**Affiliations:** 1 Laboratorio de Química de Organismos Marinos, Instituto Patagónico del Mar, Universidad Nacional de la Patagonia San Juan Bosco (UNPSJB), Puerto Madryn 9120, Chubut, Argentina; mavaro@unpata.edu.ar; 2 Laboratorio de Oceanografía Biológica, Centro Para el Estudio de Sistemas Marinos (CESIMAR), CONICET, Puerto Madryn 9120, Chubut, Argentina; barbieri@cenpat-conicet.gob.ar; 3 Laboratorio de Virología, Instituto Patagónico del Mar, Universidad Nacional de la Patagonia San Juan Bosco (UNPSJB), Puerto Madryn 9120, Chubut, Argentina; 4 Instituto Tecnológico de Chascomús, The Chascomús Technological Institute (INTECH), CONICET-UNSAM, Chascomús 7130, Buenos Aires, Argentina; ayelen@gazquez.com.ar

**Keywords:** cytokine storm syndrome, natural products, sea urchin, pigments, spinochromes

## Abstract

**Background**: Echinochrome A (EchA) is a pigment from sea urchins. EchA is a polyhydroxylated 1,4-naphthoquinone that contains several hydroxyl groups appropriate for free-radical scavenging and preventing redox imbalance. EchA is the most studied molecule of this family and is an active principle approved to be used in humans, usually for cardiopathies and glaucoma. EchA is used as a pharmaceutical drug. **Methods**: A comprehensive literature and patent search review was undertaken using PubMed, as well as Google Scholar and Espacenet search engines to review these areas. **Conclusions**: In the bloodstream, EchA can mediate cellular responses, act as a radical scavenger, and activate the glutathione pathway. It decreases ROS imbalance, prevents and limits lipid peroxidation, and enhances mitochondrial functions. Most importantly, EchA contributes to the modulation of the immune system. EchA can regulate the generation of regulatory T cells, inhibit pro-inflammatory IL-1β and IL-6 cytokine production, while slightly reducing IL-8, TNF-α, INF-α, and NKT, thus correcting immune imbalance. These characteristics suggest that EchA is a candidate drug to alleviate the cytokine storm syndrome (CSS).

## 1. Introduction

A wide scope of published research indicates that the molecule echinochrome A (EchA) is a potent free-radical scavenger, diminishing reactive oxygen species (ROS) and preventing redox imbalance [1]. In addition, EchA can enhance the immune system response and, in terms of mitochondrial functioning, helps to increase glutathione (GSH) levels [2,3,4]. In the following sections, we describe the how EchA can contribute to alleviate the Cytokine Storm Syndrome (CSS). First, we describe the pharmacological applications of EchA, and then address the mechanisms by which EchA decreases ROS and maintains redox balance. The second section details how EchA influences the immune system response. Finally, we highlight the major issues regarding CSS and examine the potential role of EchA as an agent alleviating CSS.

## 2. Echinochrome A and Its Pharmacological Applications

**Figure 1:**
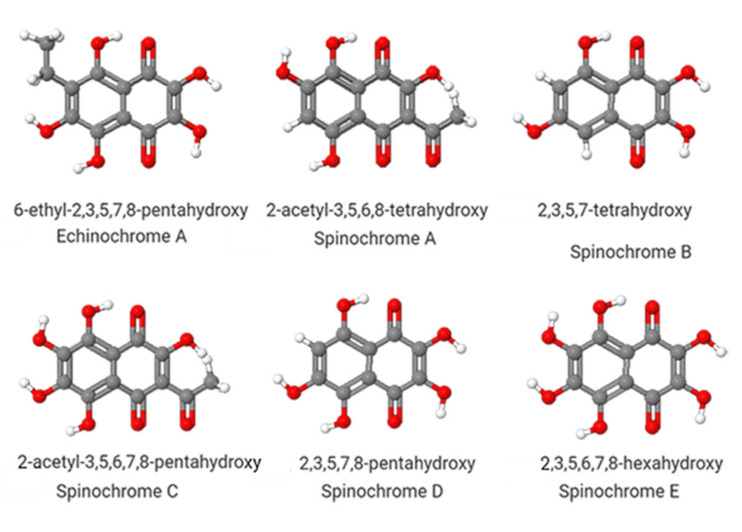
Chemical structures of 1,4-polyhydroxylated naphthoquinone derivatives from sea urchins. Created with JSME-Jmol (accessed on 9 September 2020).

Polyhydroxylated 1,4-naphthoquinones are pigments found in sea urchin shells, spines, gonads, coelomic fluid, and eggs, commonly known as spinochromes [5,6,7,8]. Quinones are known to have pharmacological properties [9]. In particular, the spinochromes contain several hydroxyl groups. These groups are appropriate for free-radical scavenging, which diminishes ROS and prevents redox imbalance [10] (Figure 1). Echinochrome A (EchA, 6-ethyl-2,3,5,7,8-pentahydroxy-1,4-naphthoquinone) is the most researched molecule of this family and is the active substance in the drug Histochrome™. This drug was developed, patented, and approved in Russia (PN002363/02-2003, EP1121929A1). This pharmaceutical preparation comprises the biologically active additive dietary supplement, Thymarin^®^, which is orally ingested (Sanitary-Epidemiological Conclusion No. 77.99.03.935.Á.000138.06.04 dated 14 June 2004, TU 9350-064-02698170-2004; RU2340216C1). The chemistry and pharmacokinetics of EchA [11] were investigated and EchA was used in orally ingested formulations [12], complying with the Lipinski Rule of five for orally available compounds (data not published). We summarize the focus of our investigation in terms of pharmacological effects in Table 1. The bioactive actions of spinochromes are numerous, indicating that spinochromes can potentially be used for several different medical conditions. We focus on the effect of EchA on the redox imbalance and on the immune system due to its impact on CSS. 

## 3. Echinochrome A and Redox Imbalance

Free radicals (superoxide, hydroxyl radicals, nitric oxide, and peroxynitrite) are destructive molecules when they are imbalanced. This imbalance generates oxidative stress that can produce different types of damage to varying degrees: protein deterioration, DNA damage, lipid peroxidation, and can even result in cell death and organ failure [35,36,37]. The human body incorporates a complex antioxidant network that fights against free radicals to resist vital biomolecular damage. The first line of defense in the antioxidant network is generated by endogenous enzymes, superoxide dismutase (SOD), catalase (CAT), and glutathione peroxidase (GPX) [38]. SOD catalyzes the dismutation of superoxide to oxygen and hydrogen peroxide. Hydrogen peroxides are key in mediating systemic signals and regulating transcription factors such as the protein complex NF-κB. This transcription factor is important in controlling inflammatory responses. Such responses include nuclear factor erythroid 2-related factor 2 (Nrf2), which stimulates the transcription of target genes, resulting in an antioxidant response [39], and protects cells from inflammation [40]. Another such response is mediated by peroxisome proliferator-activated receptors (PPAR-γ), which regulate cell metabolism and heat shock proteins [41]. CAT constitutes an important enzymatic defense as it allows for the independent control and maintenance of H_2_O_2_ in cell culture, ensuring the decomposition of hydrogen peroxide into water and oxygen [42]. GPX uses glutathione (GSH) to reduce hydrogen peroxide and other peroxides, such as hydroperoxides and xenobiotics.

The second line of antioxidant defense comprises the balance of various antioxidant compounds, such as GSH, which is partly regulated by GPX and neutralizes or eliminates free radicals into harmless redox species [38]. An ideal antioxidant compound should scavenge ROS and break down modular networks to avoid lipid peroxidation and GSH depletion [38].

EchA treatment in diabetic mice showed a glucose concentration reduction as well as a general increase in glutathione-S-transferase (GST), SOD, and CAT activities. It also evidenced an incremental increase in GSH and oxide nitrogen concentration, and improved general renal function [3]. In addition, the malondialdehyde (MDA) concentration, a generally accepted marker of lipid peroxidation, diminished when mice were treated with EchA [3]. EchA administration also showed indications of improving liver function in diabetic mice and diminishing glucose and MDA concentrations. Furthermore, there was a general increase in the levels of insulin and the activities of GST, GPx, and SOD enzymes, as well as a general increase in the GSH concentration [22]. In Wistar albino rats with diabetes mellitus, EchA treatment also resulted in a reduction in glucose and MDA levels, but simultaneously showed increases in GST, SOD, and GPx activities. GSH concentration also increased in Wistar albino rats [22]. In addition, EchA was noted as potentially comprising an alternative antiseptic remedy due to the finding that its administration to Winstar albino rats improved their liver function by counterbalancing hepatic oxidative stress through the increase in the GSH concentration, as well as of SOD, CAT GPX and GST activities, and by downregulating MDA and nitric oxide [30]. EchA applied as an antitumor treatment in Swiss albino mice with Erlich ascites tumors also resulted in increases in CAT and GST activities, as well as an increased in GSH concentration. This effectively reduced the tumor volume [33]. In hyperlipidemic rats, EchA administration showed hypolipidemic effects and a significant increase in antioxidant markers, such as GST, CAT, and GSH. Furthermore, EchA in these rats showed a reduction in MDA [31]. In postnatal rats, EchA was also observed to diminish the severity of bleomycin-induced oxidative stress in the lungs, preventing the hypertrophy of interalveolar connective tissue and peribronchial lymphoid infiltration [15]. In cardiomyocytes treated with doxorubicin, EchA produced a decrease in ROS [1], whereas spinochrome D, a structural analog to EchA, showed an increase in the ATP production, the oxygenation of cells, and increased GSH levels [1,29]. In humans, EchA treatments have resulted in improvements in glucose metabolism, inhibition of lipid peroxidation processes, and an increase in CAT activity and GSH concentration [12,21]. The increase in hydrogen peroxide by EchA generates increases in peroxisomes and mitochondria in cells, leading to an increase in CAT activity [12,21,43]. In humans, EchA showed nitric-oxide- and hydrogen-peroxide-mimicking effects in endothelial and smooth muscle vascular cells. This generated vasodilation and reduced ischemia and hypoxia [12,44]. 

**Figure 2:**
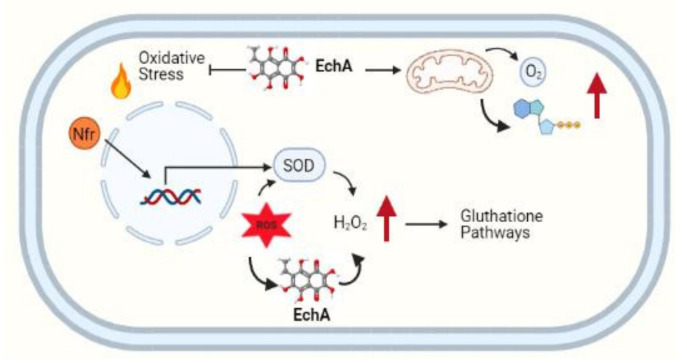
EchA mediates cellular responses, acts as a radical scavenger preventing lipid peroxidation, improves mitochondrial activity, and activates the glutathione pathway, diminishing the overall ROS imbalance. Created with BioRender.com (accessed on 9 September 2020).

Overall, the effects of EchA appear to implicate its potential role as an ideal antioxidant. This is due to its ability to neutralize ROS, chelate metals, and serve as an intracellular messenger, promoting hydroperoxides decomposition, avoiding lipid peroxidation, and increasing GSH production. As a consequence, mitochondria perform better, and the oxygen and ATP levels increase in the cell [12,21,43,44]. In summary, EchA acts as a primary antioxidant by attacking superoxides, but also works as a secondary antioxidant by increasing the hydrogen peroxide level, mimicking the SOD enzyme, and triggering the GSH pathway (Figure 2).

## 4. Echinochrome A and Immune System Response

**Figure 3:**
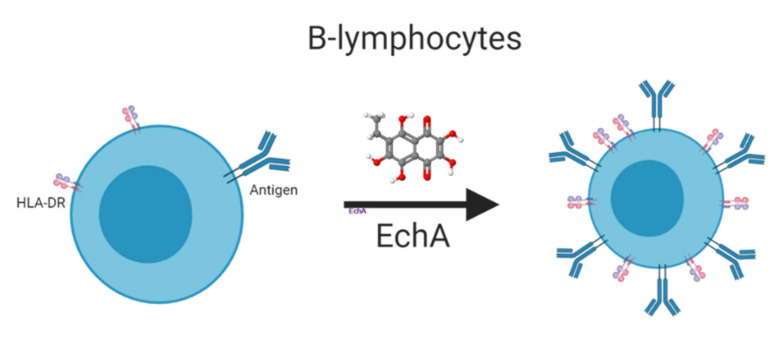
EchA may increase human leukocyte antigens (HLA-DR) in B lymphocytes, increasing antigen processing and presentation. Created with BioRender.com.

**Figure 4:**
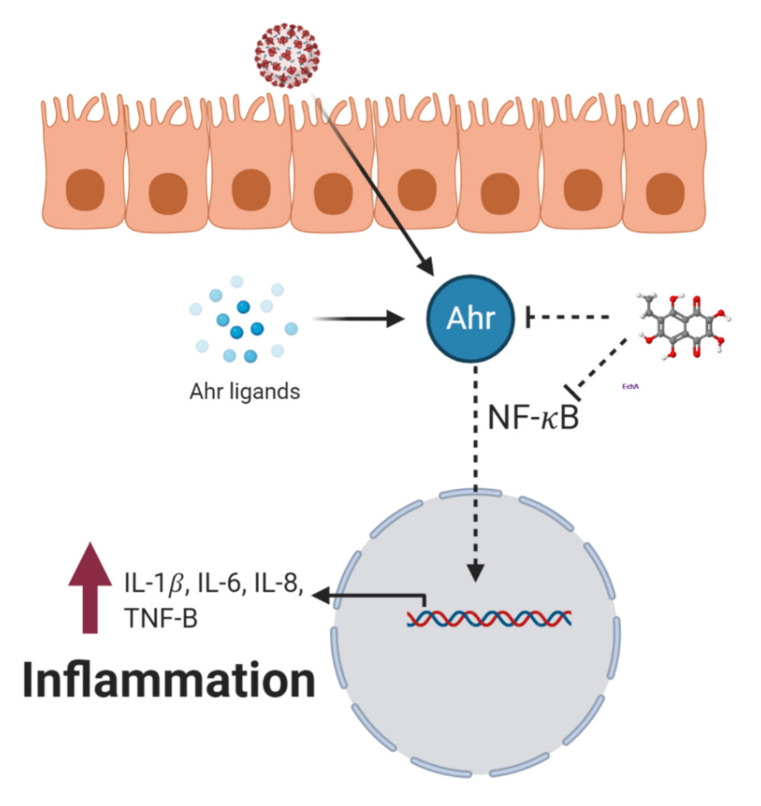
Proposed inhibition of NF-κB pathway by EchA through aryl hydrocarbon receptor (Ahr). Created with BioRender.com.

As free radicals play an important role in the regulation of cytokines, growth factors, cell signaling, immunomodulators, etc. [45], maintaining redox imbalance also affects the regulation of these pathways. EchA is able to induce changes in the T-helper link and increase the number of B-lymphocytes and human leukocyte antigens (HLA-DR), triggering the cooperation among immune cells, as well as increasing antigen processing and presentation [12] (Figure 3). This relationship between EchA and the immune response has been observed in studies on the influenza virus using EchA as an adjuvant in antisera preparations [46], as well as using EchA treatments applied orally or by injection [12]. In addition, EchA regulates the immune response by promoting the generation of regulatory T cells (Tregs). These Tregs cells play a critical role in inhibiting T cell proliferation and cytokine production. Tregs control the immune response to antigens and help prevent autoimmune diseases, without affecting the Th1 or Th2 populations. As Tregs functions are key immunomodulators regulating the activation of other Th cells, EchA may contribute to correct immune imbalances by inducing the Tregs population [16]. As EchA acts as an agonist for aryl hydrocarbon receptors (Ahr), it is able to switch the immune response toward Th1 and inhibit the NF-κB pathway (Figure 4) [12]. It was shown that EchA can not only highly decrease proinflammatory cytokines IL-1β and IL-6, but can also slightly reduce IL-8, TNF-α, INF-α, and NKT [12] (Figure 4), helping with the decrease in cellular inflammation. Generally, M1 macrophages mediate excessive and persistent pro-inflammatory effects, and M2 macrophages contribute to the regeneration and resolution of inflammatory tissues. EchA was shown to diminish the secretion of TNF-α derived from M1 macrophages in a dose-dependent manner and to increase the level of basal secretion of IL-10-inducing M2 macrophages. This change in the immune response leads to a decrease in cellular inflammation [16]. In addition, the hydrogen peroxide produced by EchA in the bloodstream induces the overexpression of a co-activator of the PGC-1α, PPAR-γ receptor [21,43,47], up-regulating Fas (CD95+) and Fas-ligand (CD178+) [48], primarily in the activated cells in the immune system, such as Th1 and NK cells [48,49]. EchA can boost the immune response because it intensifies the intercellular cooperation of immune cells and increases the process and presentation of antigen.

## 5. Cytokine Storm Syndrome

The term cytokine storm syndrome (CSS) has received much attention, both in popular media and scientific literature during the last twelve months due to CSS being a common occurrence in COVID-19 patients with comorbidities [50,51,52,53,54,55]. CSS is a systemic inflammatory response mediated by cytokines resulting in overwhelming systemic inflammation, hemodynamic instability, multiple organ dysfunction, sepsis, fever, hyperferritinemia, and, in many cases, death [56,57,58]. The cytokines function comprises intercellular signaling and communication. Cytokines are small proteins that, by binding to the receptor, trigger a variety of responses, including immune and inflammatory responses [57]. As cytokines are part of the innate immune system, CSS is not a disease in and of itself, but rather comprises a hyperactive response of the immune system to infectious agents, malignant tumors, rheumatic diseases, iatrogenic injury, and immunotherapeutic drugs; however, infection agents like viruses and bacteria are the most common cause [54,55]. With CSS, the release of cytokines is injurious to the host cells and damages the organs, which is sometimes irreparable [58,59,60]. CSS is triggered by the release cytokines interferon (IFN)-γ, tumor necrosis factor (TNF), and interleukin (IL)-1, IL-6, and IL-18 [61,62,63,64]. Despite the significant amount of research on this topic, the pathogenesis of CSS is not yet fully understood. Still, it appears to be generally agreed upon that there is an imbalance in proinflammatory and anti-inflammatory agents and pathways during CSS. As a result, the majority of the treatments comprise immunosuppression together with treatment seeking to control the initial trigger of the production of CSS [57,59,65]. However, it is the combination of pharmaceutical, nutraceutical, and adjunctive treatments that might help in the fight against CSS. When CSS occurs, an inability to resolve the inflammation arises, causing catastrophic damage [66]. As a result, the ability to sufficiently regulate the inflammatory response may prevent the body from systemic inflammation. Cytokine balance needs to be restored to effectively resolve the inflammation caused by environmental influences, such as infection agents. 

During CSS, release of cytokines increased, specifically IL-1 β, IL-6, and TNF-α; all of the cytokines comprise proinflammatory factors [57,67] leading to an increase in ROS production by the mitochondria [68]. In turn, ROS activates signal transduction pathways, such as NF-κB, which also stimulate the production of pro-inflammatory cytokines, generating increased inflammation and redox imbalance [66]. GSH is the most important intracellular system in scavenging these ROS [69,70]. However, the presence of ROS overproduction often leads to GSH depletion [69], which has serious consequences in the protective functions of organs, often leading to an immunocompromized state due to the impairment of the functions of lymphocytes, macrophages, and neutrophils. In some cases, GSH depletion triggers lymphopenia through the activation of the apoptotic cascade [71,72]. In this context, to counterbalance oxidative stress, it is most important to prevent GSH depletion and its negative consequences during CSS.

## 6. Echinochrome A and Cytokine Storm Syndrome

Although no specific treatment exists for CSS, immunosuppression, anti-inflammatory and antioxidant treatments have been suggested to be suitable approaches in reducing and modulating CSS, and the resulting hyper-inflammation and GSH depletion (Table 2). IL-1- and IL-6-inhibiting agents were found to diminish CSS by preventing inflammation. Anakinra is an IL-1-inhibiting agent that is able to block IL-1 by competitively inhibiting their binding to IL-1 receptors [73,74]. Although other agents may inhibit IL-1, such as canakinumab and rilonacept, further research is needed [54]. Tocilizumab is an IL-6-inhibiting agent (monoclonal antibody) that binds to soluble and membrane-bound IL-6 receptors, and was proven to be an efficient therapy to alleviate CSS when combined with T cell engaged therapy [75]. However, this was shown not to apply to every case of CSS [76]. Curcumin was shown to suppress multiple cytokines in conditions associated with CSS. Still, curcumin is poorly absorbed from the intestinal tract, hence, requiring intravenous formulations [77]. As EchA is able to reduce the production of pro-inflammatory cytokines IL-1β and IL-6, to increase anti-inflammatory cytokines such as IL-10 [12], and to diminish TNF-α secretion [19,28], EchA may help to restore the proinflammatory and anti-inflammatory balance and, as such, aid in the alleviation of CSS.

The counterbalancing of oxidative stress is crucial in CSS [50] to prevent GSH depletion and its disastrous consequences [69]. Counterbalancing oxidative stress is also important in avoiding signal transduction pathways that stimulate pro-inflammatory cytokines production [66]. As a result, treatments that are able to reduce ROS can help with treating CSS. The use of the enzyme CAT was proposed as CAT was to have an anti-inflammatory effect by regulating cytokine production and providing protection against oxidative injury in human leukocytes and alveolar epithelial cells [78]. This enzyme is safe and commonly used as a food additive and dietary supplement. N-acetylcysteine (NAC) was also suggested for use in preventing CSS [66], as it is a prodrug to L-cysteine and, in turn, L-cysteine is a precursor to GSH. NAC was shown to inhibit the production of pro-inflammatory cytokine IL-6 in H5N1-infected lung cells [79]. However, current clinical evidence indicates that its bioavailability is low [80]. Considering all these factors, EchA may play a crucial role in CSS treatment as one of its most important characteristics is that it can increase GSH metabolism and, as such, prevent the GSH depletion found in CSS patients [3,12,21,22,30,43,44].

In summary, EchA may provide multiple impacts including the reduction in inflammation, ROS scavenging, and the induction of GSH pathways. In addition, EchA’s pharmacological activities, low toxicity level [5], and high bioavailability according to Lipinski’s rule of five (data unpublished) all strongly support the view that EchA can potentially be used in CSS therapy. 

## Figures and Tables

**Table 1:** Pharmacological effects of spinochromes.

Spinochrome	Effect	Experimental model	Reference
Spinochrome D, E	Antiallergic effect	Guinea pigs	[13]
Echinochrome A	Antigen-stimulated degranulation in cellular systems	RBL-2H3 cells	[14]
Echinochrome A	Antioxidant on bleomycin-induced pulmonary fibrosis	Rats	[15]
Echinochrome A	Inflammatory bowel disease and correcting immune system imbalance	Mouse	[16]
Echinochrome A	Cardioprotective activity	Human	[17]
Echinochrome A	Acetylcholinesterase inhibition	H9c2 and A7r5 cells	[18]
Echinochrome A	Antistress effect	Bone marrow cells in SHK mice	[19]
Echinochrome A	Amelioration of intraocular inflammation (uveitis) caused by endotoxins	Lewis rats	[20]
Echinochrome A	Decreased risk of atherogenesis and improvement of glutathione metabolism	Human	[21]
Echinochrome A	Cardiomyocyte protection against toxic agents	Rat cardiac myoblast H9c2 cells and isolated rat cardiomyocytes.	[1]
Echinochrome A	Reduction in diabetic complications in liver	Wistar albino rats	[22]
Echinochrome A	Improvement of the musculoskeletal system and the metabolism of lipids and proteins in both types of diabetes mellitus	Wistar albino rats	[23]
Echinochrome A	Improvement in the renal function and ameliorating renal histopathological	Winstar albino rats	[3]
Echinochrome A	Enhancement in exercise capacity	Sprague–Dawley rats	[24]
Echinochrome A	Prevention and/or deceleration of PD-like neurodegeneration	Rats	[25]
Echinochrome A	Hepatoprotective effect against intrahepatic cholestasis induced by toxic agents	Rats	[26]
Echinochrome A	Enhancing cardiomyocyte differentiation	Mouse embryonic stem cells	[27]
Echinochrome A	Potentiating the effectiveness of antitumor therapy	Ehrlich ascites carcinoma model	[28]
Echinochrome A	Cardioprotective against the cytotoxicity of doxorubicin	Human cardiomyocyte cell line (AC16) and human breast cancer cell line (MCF-7)	[29]
Echinochrome A	Liver antiseptic	Albino rats	[30]
Echinochrome A	Improvement in lipid profile, liver functions, kidney functions, and antioxidant markers	Rats	[31]
Echinochrome A	Cardioprotective against the cytotoxicity of doxorubicin	AC16 human cardiomyocyte cells	[4]
Echinochrome A	Hypolipidemia in obesity	Rats	[31]
Echinochrome A	Prevention of atherosclerotic inflammation, SOD3 mimetic, improves the response of the immune system	Human	[12]
Echinochrome A	Protective effects on the extracellular matrix of vocal folds in	Ovariectomized rats	[32]
Echinochrome A	Antitumor activity, decreases lipid peroxidation, and improves antioxidant status	Ehrlich ascites carcinoma tumor model in mice	[33]
Echinochrome A	Anti-inflammatory effect	Rats	[34]

**Table 2:** Proposed treatments for CSS.

Treatment	Effect in CSS	Reference
**Anakinra**	IL-1-inhibiting agent	[73]
**Canakinumab**	IL-1-inhibition agent	[54]
**Rilonacep**	IL-1-inhibition agent	[54]
**Tocilizumab**	IL-6-inhibiting agent	[75]
**Curcumin**	Cytokines suppressor	[77]
**Enzyme CAT**	Antioxidant agent: cytokine production regulator through the GSH pathway	[78]
**N-acetylcysteine (NAC)**	IL-6-inhibiting agent through the GSH pathway	[66]
